# High Prevalence of Constitutional Mismatch Repair Deficiency in a Pediatric T-cell Lymphoblastic Lymphoma Cohort

**DOI:** 10.1097/HS9.0000000000000668

**Published:** 2021-12-21

**Authors:** Emma Kroeze, Dilys D. Weijers, Melanie M. Hagleitner, Hester A. de Groot-Kruseman, Marjolijn C. J. Jongmans, Roland P. Kuiper, Rob Pieters, Jules P. P. Meijerink, Jan L. C. Loeffen

**Affiliations:** 1 Princess Máxima Center for Pediatric Oncology, Utrecht, The Netherlands; 2 Department of Genetics, University Medical Center Utrecht, The Netherlands

## Abstract

This study describes the clinical characteristics of a complete Dutch T-cell lymphoblastic lymphoma (T-LBL) cohort, including second primary malignancies and comorbidities. We show that over 10% of patients in this complete T-LBL cohort have been diagnosed with a cancer predisposition syndrome (CPS), consisting almost exclusively of constitutional mismatch repair deficiency (CMMRD). The clinical characteristics of sporadic T-LBL patients were compared with T-LBL patients that have been diagnosed with CMMRD. This shows that disease presentation is comparable but that disease localization in CMMRD patients might be more localized. The percentage of CPS seems reliable considering the completeness of the cohort of Dutch T-LBL patients and might even be an underestimation (possibility of undiagnosed CPS patients in cohort). As the frequency of an underlying predisposition syndrome among T-LBL patients may be underestimated at present, we advocate for screening all pediatric T-LBL patients for the presence of germline mutations in mismatch repair genes.

## INTRODUCTION

T-cell lymphoblastic lymphoma (T-LBL) is a subtype of non-Hodgkin lymphoma (NHL) that arises from the malignant transformation of immature T-cells, similar to T-cell acute lymphoblastic leukemia (T-ALL). T-LBL is mainly characterized by massive infiltration of blasts in the mediastinum and lymph nodes (LNs), often accompanied by pleural and pericardial fluids. T-LBL typically presents as extramedullary disease and by definition with fewer than 25% blasts in the bone marrow (BM).^1^ In the past, T-LBL patients were mainly stratified according to Murphy stage, which is determined by disease dissemination and the revised international pediatric NHL staging system.^2-4^ Even though T-LBL and T-ALL are thought to be closely related,^5^ extensive analyses have led to improved understanding of the biology of T-ALL, whereas similar efforts for T-LBL are still scarce. The lack of understanding of T-LBL biology and origin hampers the development of prognostic markers as well as new therapeutic treatment strategies. Nevertheless, recent studies have made progress in increased understanding of the biology of T-LBL.^6^ Childhood T-LBL seems to be associated with a high occurrence of second primary malignancies after treatment of NHL.^7^ This last study revealed a relatively high number of T-LBL patients in a selected cohort of NHL cases who developed second primary malignancies (69/189).^7^ This finding is a strong indicator that T-LBL in various patients may arise from an underlying cancer predisposition syndrome (CPS). However, this study concerned a selected cohort and therefore does not answer the question what the estimated prevalence of tumor predisposition syndromes in an unselected cohort of T-LBL patients will be. Even though an association between constitutional mismatch repair deficiency (CMMRD) and T-LBL is known, to our knowledge, there are no reported studies concerning the percentage of CMMRD patients in an unbiased T-LBL cohort.

In this study, we describe a complete and unselected T-LBL cohort based on clinical presentation of disease, as well as second primary malignancies and comorbidities. We found an exceptionally high percentage of CMMRD patients in the complete cohort of T-LBL patients. Subsequently, we compared the clinical characteristics of T-LBL patients with CMMRD to the sporadic T-LBL cases in the cohort.

## METHODS

### Patients and study design

This retrospective, multicenter cohort study presents the clinical characteristics from 88 pediatric T-LBL patients (between 0 and 18 y). Patients were diagnosed between January 2007 and September 2020 and treated according to the European Intergroup EURO-LB02 (EURO-LB02) protocol in pediatric oncology centers in the Netherlands. All patients or patients’ guardians signed informed consent and the current study was approved by all institutional ethic committees (19-140/C).

### Data collection

All T-LBL patients diagnosed in the Netherlands between January 2007 and September 2020 were included through the complete Dutch Childhood Oncology Group registration and data were collected in a retrospective manner from patients’ files. The following data were recorded at diagnosis for all patients: gender, date of birth, and date of diagnosis. Additionally, date of complete remission (CR), outcome, tumor stage (Murphy), second malignancies, and presence of a CPS determined by specific germline pathogenic variants were recorded. If there was no reason for suspicion of a CPS, patients have not been screened for the presence of germline pathogenic variants. In addition, hematological values and radiology results have been recorded, including leukocyte counts (normal range 5–10 × 10^9^/L), thrombocyte counts (normal range 150–450 × 10^9^/L), serum lactate dehydrogenase levels (normal range 150–450 U/L), mediastinal enlargement (as established by x-ray or computed tomography [CT]), nodal involvement (as established by CT or ultrasound) including head, neck, or supraclavicular (HNS) area, hilar, intra-abdominal, axillary, and inguinal LNs, the presence of pleural or cardiac effusion, hepatomegaly and/or splenomegaly (as established by abdominal sonogram), BM involvement (≥5% cytomorphological blasts) including percentage, and presence of blasts in the central nervous system (CNS) measured in the cerebral spinal fluid (CSF) (CNS status). CNS1 is defined as ≤5 white blood cells (WBCs)/µL CSF fluid without leukemic blasts, CNS2 as ≤5 WBC/µL CSF fluid with identifiable leukemic cells, and CNS3 as >5 WBC/µL CSF fluid with identifiable leukemic cells, intracerebral or intraspinal masses, or cranial nerve involvement.

### Statistics

Overall survival was calculated from date of initial diagnosis to date of death or date of last known follow-up. Statistical significance between values at diagnosis was analyzed by using the Mann-Whitney test or Fisher exact test (Graphpad prism v8, CA).

## Results

### Clinical characteristics of T-LBL patients

The clinical characteristics of 88 T-LBL patients are summarized in Table 1. The median age at diagnosis was 9 years (range, 0–18 y) with a male to female ratio of 1.7:1 (63% males). The mediastinum was enlarged in 94% of the T-LBL. Sixty-six percent of the patients presented with enlarged LNs, predominantly located in the HNS regions (53%). Half of the patients presented with pleural effusion (52%) and about one-third (32%) of the T-LBL patients presented with hepatomegaly. Splenomegaly was evident in 10% of the patients, mostly in combination with hepatomegaly.

**Table 1.** Clinical Characteristics of T-LBL Patients With and Without CMMRD.

Characteristics	Total T-LBL, n = 88	T-LBL Sporadic,^*a*^ n = 79	T-LBL CMMRD, n = 8
Age, y, median (range)	9 (0–18)	9 (0–18)	10 (0–16)
Sex, n			
Male	55	50	5
Female	33	30	3
Localizations, n (%)			
Mediastinal enlargement	83 (94)	75 (94)	8 (100)
LN involvement	58 (66)	55 (68)	3 (38)
HNS lesion	47 (53)	45 (56)	2 (25)
Pleural effusion	46 (52)	42 (52)	4 (50)
Hepatomegaly	28 (32)	27 (34)	1 (13)
Splenomegaly	9 (10)	9 (11)	0 (0)
Bone marrow involvement, n (%)			
1%–4%	15 (17)	15 (19)	0 (0)
≥5%	7 (8)	7 (9)	0 (0)
CNS status, n (%)			
CNS1	66 (85)	58 (84)	8 (100)
CNS2	9 (12)	9 (13)	0 (0)
CNS3	2 (3)	2 (3)	0 (0)
Missing	11	11	0
Hematological, median (range)			
Leukocytes (count × 10^9^/L)	8.1 (1.6–28)	8.3 (1.6–28)	7.3 (5.2–20.3)
Thrombocytes (count × 10^9^/L)	335 (84–645)	342 (84–645)	292 (243–560)
LDH (U/L)	546 (131–2575)	540 (131–2575)	683 (265–1143)
Second malignancy, n (%)	8 (9)	2 (2.5)	6 (75)
Overall survival, %	83	86	50

^*a*^The Fanconi Anemia patient was not included in these results.

CMMRD = constitutional mismatch repair deficiency; CNS = central nervous system; HNS = head, neck, or supraclavicular; LDH = lactate dehydrogenase; LN = lymph node; T-LBL = T-cell lymphoblastic lymphoma.

Twenty-five percent of the T-LBL patients presented with >1% blasts in their BM (range, 1%–16%) with 8% of the patients having >5% blasts. Two patients (3%) had CNS3, and 9 patients (12%) were diagnosed with CNS2. Sixty-six patients (85%) had no evidence of CNS involvement (CNS1) at disease diagnosis. For 11 patients, this data was either missing or their treatment had started prior to a diagnostic lumbar puncture due to a poor clinical condition of the patient. Median hematological values at diagnosis were within the physiological ranges (Table 1).

### Cancer predisposition syndromes in T-LBL patients

A total of 10.2% of the T-LBL patients (9/88) in our cohort have been diagnosed with a CPS based on the presence of germline pathogenic mutations: CMMRD in 8 patients and Fanconi anemia (FA) in 1 patient. CMMRD is caused by biallelic germline mutations in one of the mismatch repair (MMR) complex genes including *MLH1*, *MSH2*, *MSH6*, or *PMS2*. The CMMRD patients in this cohort had mutations that affected *PMS2* (5/8) or *MSH6* (3/8) (Table 2). All mutations were unique, and most patients had compound heterozygous MMR gene mutations (6/8). Two patients with consanguineous parents presented with homozygous mutations in *PMS2* and *MSH6*, respectively. The patient with FA had a homozygous deletion (del67G) in the *FANCC* gene, a Dutch founder mutation leading to a frameshift and a premature stop.

**Table 2.** Clinical Characteristics of Patients With a CMMRD During Their T-LBL.

Case	Sex	Gene	Variant mRNA	Variant Protein	Exon	Consanguineous Parents	Age CMMRD Diagnosis	Malignancies (Age)	Received Treatment Prior to T-LBL Diagnosis	T-LBL Localizations	BM%	CNS Status	Status	Benign Tumors	Opportunistic Infections
1	M	*PMS2*	c.904_911del	p.Val302Thrfs*4	9	No	5	Medulloblastoma (4)	COG ACN0332 w/RT	Mediastinum	0	CNS1	Death in induction T-LBL		Pulmonary aspergillosis
			c.1882C>T	p.Arg628*	11			DLBCL (8)		Jugular LN					
								T-LBL (10)	SKION B-NHL Ritux (arm B) 2008						
2	M	*PMS2*	c.137G>T	p.Ser46Ile	2	No	8	T-LBL (8)	—	Mediastinum	0	CNS1	T-LBL CR2	Low-grade adenomas	
			c.247_250dup	p.Thr84Ilefs*9	3			Relapse T-LBL (10): reinduction chemotherapy followed by allogeneic SCT		Pericardial effusion					
										Axillary LN					
										Intra-abdominal LN					
										Testis					
3	F	*PMS2*	c.943C>T (hom)^*b*^	p.Arg315*	9	Yes	14	T-LBL (14)	—	Mediastinum	0	CNS1	T-LBL CR1	Benign pilomatrixoma	Pulmonary aspergillosis
								Squamous carcinoma skin (17)		Pleural effusion					
										Cervical LN					
4	M	*MSH6*	c.651dup	p.Lys218*	4	No	15	T-LBL (16)	—	Mediastinum	0	CNS1	T-LBL CR1	Low-grade adenomas	
			c.3957dup	p.Ala1320Serfs*5	9			Cecum carcinoma (19)							
5	F	*PMS2*	c.989-296_1144+706del	p.Glu330_Glu381del	10	No	10	Glioblastoma multiforme (8)	COG ACNS0126 w/RT	Mediastinum	0	CNS1	Death in maintenance T-LBL		
			c.319C>T	p.Arg107Trp	4			Glioblastoma multiforme (10)	COG ACNS0126 w/RT	Pleural effusion					
								T-LBL (10)							
6	M	*PMS2*	Deletion	Protein truncation	2	No	13	DLBCL (9)	SKION B-NHL 2008	Mediastinum	0	CNS1	Death, 7 y after T-LBL CR1	Tubular adenoma	
			Deletion	Protein truncation	5–15			Relapse or new DLBCL (13)	Autologous SCT	Cervical LN					
								T-LBL (14)		Pericardial effusion					
								Astrocytoma (20)							
								Mediastinal lymphoma, NOS (21)							
7	M	*MSH6*	c.3991C>T (hom)^*a*^	p.Arg1331*	9	Yes	6	T-LBL (0)	—	Mediastinal	0	CNS1	T-LBL CR1	Fibrohistiocytic lesion	
										Pleural effusion					
										Hepatomegaly					
8	F	*MSH6*	c.2815C>Tc.3801+1_3801+5del	p.Gln939*p.Arg1217Metfs*6	48	No	10	Atypical parieto-occipital rhabdoid tumor (4)	DCOG MMT 935 arm B	Mediastinal	0	CNS1	Death, 4 y after T-LBL CR1		Pulmonary aspergillosis
								T-LBL (6)	Autologous SCT	Pleural effusion					
								MDS (10)							

Dashes indicate that these patient had not received treatment prior to T-LBL diagnosis.

^*a*^The Fanconi Anemia patient was not included in these results.

^*b*^Homozygous mutation.

BM = bone marrow; B-NHL = B-cell non-Hodgkin lymphoma; CMMRD = constitutional mismatch repair deficiency; CNS = central nervous system; CR = complete remission; DCOG = Dutch Childhood Oncology Group; DLBCL = diffuse large B-cell lymphoma; F = female; LN = lymph node; M = male; MDS = myelodysplastic syndrome; mRNA = messenger RNA; NOS = not otherwise specified; SCT= stem cell transplantation; T-LBL = T-cell lymphoblastic lymphoma.

### Clinical presentation of T-LBL patients with a CMMRD

The age of the CMMRD patients at diagnosis of their first tumor ranged from 1 to 17 years, with a median age of 10 years (Table 2). The median age at CMMRD diagnosis was 10 years (range, 5–15 y) as well. All patients with a CMMRD had multiple café-au-lait maculae at the time of CPS diagnosis, and diagnosis was established in 5 out of 8 cases at times of their first malignancies. The lag period between diagnosis of the first tumor and CMMRD diagnosis ranged from 1 to 6 years. For 2 out of 8 cases, neurofibromatosis type 1 (NF1) was initially suspected, but mutational analysis of *NF1* turned out to be negative. CMMRD diagnoses were expected based on the development of second primary childhood tumors, malignancies in siblings, consanguineous parents, recurrent polyps in the small intestine, café-au-lait maculae, or a combination of these factors. One patient was already diagnosed with CMMRD prior to development of the first childhood tumor. The patient with FA had small café-au-lait maculae but had no signs of congenital anomalies. Prolonged thrombocytopenia combined with infectious complications led to FA diagnosis during T-LBL treatment.

All CMMRD patients in this cohort had evident mediastinal enlargements that were accompanied by pleural effusions in 5 patients and pericardial effusion in 1 patient. Four patients presented with involvement of a single or multiple LNs, often in the HNS region. One patient presented with hepatomegaly in addition to an enlarged mediastinum and pleural effusion, but none of the patients had splenomegaly. More extensive spread of disease was observed in 1 patient only. None of the CMMRD patients had evidence of malignant blasts in the BM, peripheral blood, or CNS compartments.

### Second primary malignant neoplasms

Seventy-five percent (6/8) of the CMMRD patients had a history of either preceding or following malignancies, so far. T-LBL was the first malignancy in 4 patients. One patient had 1 malignancy preceding T-LBL diagnosis, 3 patients had a history of 2 prior malignancies before T-LBL diagnosis, including a possible relapse in 1 patient (Table 2). Half of the CMMRD patients who developed a T-LBL had received previous chemotherapeutic treatment. Of the 6 patients who went into CR after the end of T-LBL treatment, 1 patient relapsed, whereas 2 other patients developed new malignancies. Two patients remained cancer-free so far, although still having a relative short follow-up. Five of the CMMRD patients also presented with benign tumors.

### Comparison of CMMRD and sporadic T-LBL patients

The clinical presentation of T-LBL for CMMRD and sporadic patients were comparable, but CMMRD patients less frequently presented with 1 or multiple enlarged LNs than sporadic T-LBL patients (*P* = 0.1179). Furthermore, none of the CMMRD T-LBL patients had BM involvement at diagnosis, compared with 28% of the sporadic T-LBL patients who presented with >1% blasts in their BM and 9% having >5% blasts. Similarly, all CMMRD T-LBL patients had CNS1, whereas 3% (2/79) of the sporadic T-LBL patients had CNS3 and 11% had CNS2 (9/79). This may indicate that the T-LBL manifestation for CMMRD patients is more localized compared with sporadic T-LBL patients.

The most significant difference between CMMRD and sporadic T-LBL patients was the development of second primary malignant neoplasms (6/8 compared with 2/79) (*P* < 0.0001)). All malignancies that the CMMRD patients had developed are described in Table 2. The second neoplasms that developed in the sporadic T-LBL patients were a T-ALL in 1 patient and an acute myeloid leukemia (AML) in the other patient. The T-ALL occurred 7 years after initial T-LBL diagnosis, whereas the AML developed during maintenance treatment of the T-LBL. Both patients are still alive.

### Treatment and infections of CMMRD patients

The treatment response of the CMMRD T-LBL patients was favorable, 6 out 8 patients (75%) achieved CR. One patient died during induction therapy due to pulmonary aspergillosis. This patient was heavily pretreated for a diffuse large B-cell lymphoma 2 years prior to development of T-LBL and a medulloblastoma 6 years earlier. The other patient who did not achieve radiological CR died of metastatic cerebral disease during maintenance treatment of the T-LBL. One patient who achieved initial CR, relapsed during maintenance treatment. This patient was rescued with reinduction chemotherapy, followed by allogeneic stem cell transplantation (allo-SCT) (follow-up time 7 y after allo-SCT). The CMMRD patients for whom this information was available all received (n = 4) 150%–200% mercaptopurine (6-MP) and methotrexate during maintenance treatment (200% is maximum dosage according to EURO-LB02 protocol). In general, treatment was tolerated well in the CMMRD patients, without significant delay of chemotherapy. However, 3 out of 8 CMMRD patients developed pulmonary aspergillosis infection during T-LBL treatment, which was fatal for 1 patient.

## DISCUSSION

This study describes the clinical characteristics of a complete cohort of T-LBL patients in the Netherlands, in which 10.2% of the patients have been diagnosed with an underlying CPS, almost exclusively CMMRD. It will be important to study whether this percentage will be similar for other countries. However, the percentage of consanguinity in the Netherlands is similar compared with most other European countries and most mutations were compounds heterozygous. In addition, no particular founder mutations have been described for *PMS2* or *MSH6* in the Netherlands, only for the total Western population.^8^ However, 2 patients in our cohort have mutations that are recurrently found in the Caucasian population or CMMRD families in the Netherlands (unpublished data Leiden University Medical Center, Leiden, The Netherlands). These are the *PMS2* c.137G>T and *MSH6* c.651dup mutations, respectively.^8^ The patient with FA had a homozygous deletion (c.67delG) in the *FANCC* gene, which is a Dutch founder mutation leading to a frameshift and a premature stop.^9^

The CMMRD-associated T-LBL patients in our study appeared to show more localized disease, but these findings need to be validated in a larger cohort of CMMRD-associated T-LBL patients in the future. In addition, 38% (3/8) of the CMMRD patients developed a pulmonary aspergillosis. If we compare this data to the complete EURO-LB02 study, only 1% (3/233) of the T-LBL patients had developed a pulmonary aspergillosis during treatment that was fatal for 1 patient.^2^ However, numbers of our study are small and some of the patients were heavily pretreated with chemotherapy. Another shared characteristic among CMMRD patients was the presence of second primary malignancies and/or benign tumors. One sporadic T-LBL patient developed a second primary malignancy (T-ALL) 7 years after initial T-LBL diagnosis but was not screened for potential germline mutations associated with CPSs. In addition, another sporadic T-LBL patient developed an AML during maintenance treatment. Given the rarity of the development of multiple consecutive childhood malignancies in sporadic cancer patients, this is a strong indicator for the presence of a potential underlying CPS. Including these patients would make the total percentage of patients with a CPS in our cohort 12.5%.

The diagnosis of a CPS is highly dependent on the alertness of the treating physician and is often missed at time of diagnosis of the first (childhood) malignancy.^10^ Earlier CMMRD diagnosis could result in surveillance strategies for the patient and potential siblings in order to diagnose new tumors at an earlier stage. There are also indications that patients with CMMRD display increased chemoresistance, especially against thiopurines.^11,12^ This might be in concordance with the finding that CMMRD patients for whom this information was available in this cohort, all received 150%–200% 6-MP and methotrexate during maintenance treatment.^2^ Diagnosis of CMMRD at an earlier stage could result in possible adjustment of chemotherapeutic treatment strategies when needed.

A scoring system by the care for CMMRD (C4CMMRD) consortium has been proposed for which patients with a score of 3 or higher have an indication for CMMRD testing.^13^ The presence of a T-cell lineage NHL alone has been assigned 2 points. Therefore, the presence of any other feature in this classifier would make T-LBL patients suitable for genetic testing. This shows the great importance to closely look for café-au-lait maculae in children with T-LBL but also to request extensive information about carcinomas from the Lynch syndrome spectrum in relatives, as well as other features mentioned in this proposed scoring system.^13^ As the frequency of an underlying predisposition syndrome among T-LBL patients may be underestimated at present and additional features might be missed, we advocate for screening all pediatric T-LBL patients for the presence of germline mutations in MMR genes.

## AUTHOR CONTRIBUTIONS

EK conducted the study and wrote the manuscript. JPPM and JLCL designed the study and supervised the study. DDW, MMH, HGK, MCJJ, RPK and RP contributed to the study design, provided data, and approved the final version of the paper.

## DISCLOSURES

EK was sponsored by Stichting Kinderen Kankervrij (KiKa-393). All the other authors have no conflicts of interest to disclose.
